# Between sexual autonomy and protection: siloed capacity and the drift towards risk regulation in *A Local Authority v ZX* [2026] EWCOP 3

**DOI:** 10.1093/medlaw/fwag025

**Published:** 2026-07-10

**Authors:** Megan McSherry

**Affiliations:** Department of Law, University of Manchester Law School, Manchester M13 9PL, United Kingdom

## I. Introduction

The current test for sexual capacity was articulated by the Supreme Court in *A Local Authority v JB*.^1^ It requires, first, that P understands the nature and character of the sexual act; secondly, that P understands that the other person must give consent before and throughout the act; thirdly, that P can say no to the act; and lastly that P can understand the reasonably foreseeable consequences of the act, which can include pregnancy and sexually transmitted infections.^2^  *JB* widened the test of sexual capacity, creating an approach centred on consent and acknowledging that sexual relations inherently involve another autonomous person whose rights and bodily integrity must be protected.^3^ It signified a substantial shift, as the Mental Capacity Act 2005 (MCA) has traditionally been framed as a statute concerned with protecting and empowering P, rather than managing risks posed by P to other parties.

The case of *A Local Authority v ZX*^4^ builds on the developments in *A Local Authority v JB* and reveals the practical and doctrinal shortcomings of the functional approach under the MCA.^5^ Where decision-making domains are closely interrelated, the MCA’s functional approach requires the assessment of each in isolation,^6^ which can produce contradictory or incoherent capacity findings that misrepresent the reality of how decisions are made. Furthermore, where P poses a serious sexual risk to others, the functional test risks being distorted by safeguarding concerns in ways that extend the MCA beyond its intended purposes, producing overprotective outcomes that the Act’s structure cannot adequately scrutinize.

This commentary examines and critiques the litigation history of *ZX* across three stages: at first instance, in the Court of Appeal, and at the remitted hearing. I argue that the doctrinal approach to sexual capacity under the MCA lacks coherence and that *ZX* exposes the necessity for a more context-sensitive and relational interpretation of the functional test.

## II. Factual background

ZX was an 18-year-old man with diagnoses of attention deficit hyperactivity disorder (ADHD), conduct disorder, and attachment difficulties.^7^ He and his brother, YX, were removed from their birth parents following evidence of neglect and significant physical injuries. They were adopted together in 2007.^8^ The proceedings were triggered by ZX having to leave his care placement for children and the resulting need to plan for his transition into adulthood.^9^

In 2012, the brothers came to the attention of the local authority following disclosures of harmful sexual behaviours between each other and towards animals.^10^ It was considered that sexually dysfunctional attitudes had developed from their early experiences. ZX subsequently disclosed sexual thoughts characterized by violence and coercion.^11^ In 2022, he made allegations within therapy that the adoptive parents had perpetrated regular sexual abuse of both brothers from when ZX was 8 years old. These claims prompted a police investigation, which led to no charges.^12^ Much of the evidence about the risk ZX posed was derived from his own self-reporting during therapy sessions, a factual difficulty that HHJ Burrows at first instance expressly acknowledged.^13^

ZX’s most recent placement was marked by several concerning incidents directly relevant to the proceedings. He absconded to meet someone he had encountered online and was stranded overnight.^14^ He messaged a younger resident at his previous placement requesting indecent images, which resulted in police involvement.^15^ He formed a relationship with a former support worker without appreciating the professional concerns this raised. Additionally, he remained in contact with former residents with evidence of messages in which ZX and a former resident encouraged each other to show their genitals.^16^ These incidents form the factual foundation for the contact capacity assessment at the remitted hearing.

## III. Legal issues

Two features of the legal trajectory of *ZX* are especially significant. First, the appeal in *ZX* succeeded not because the facts were disputed but because the legal approach to assessing capacity to engage in sexual relations was found to be flawed. Secondly, the outcome of remission that ZX had capacity for sexual relations but lacked capacity for contact with others generated a tension between risk management and the promotion of autonomy that sits at the heart of the two critiques advanced in this commentary.

### A. Sexual capacity, risk, and impulsivity

At first instance, HHJ Burrows held that ZX lacked the capacity to engage in sexual relations, placing considerable emphasis on ZX’s impulsivity, inability to regulate behaviour, and difficulties making safe decisions in the moment. HHJ Burrows heard from Dr Ince that ZX’s ADHD produced extreme impulsivity, and that this impulsivity removed his ability to use and weigh information ‘in the moment’.^17^ That is, at the point of refusal itself, rather than only in the abstract setting of a capacity assessment. The concern was not whether ZX could articulate an understanding of consent when asked, but whether, ‘because of his mental disorder, he would not be able to use and weigh (or process) his understanding of their right to refuse being respected’.^18^

In order for HHJ Burrows to reach a conclusion, he must be satisfied that, on the basis of all the evidence, ZX was not able to meet the *JB* test.^19^ Consequently, in applying the test of *JB*, HHJ Burrows stated the question he had to ask himself about ZX was:

If ZX is engaged in sexual activity or is in a situation where sexual activity is anticipated/expected by him with a person and consent from the other party is either not forthcoming or is withdrawn will ZX be able to make a capacitous decision about whether to stop that sexual activity accordingly?^20^

Weighing this evidence, HHJ Burrows concluded that the presumption of capacity of ZX to engage in sexual relations was displaced: ZX’s mental disorder meant that he was unable to use and weigh relevant information concerning his would-be or actual sexual partner’s refusal to, or withdrawal of, consent in real time.^21^

This reasoning raises a significant doctrinal issue concerning the scope of the functional test of the MCA, as the judgment appeared to blur the distinction between an inability to make a decision and an inability to implement or control behaviour associated with that decision. In effect, impulsivity and the inability to control sexual urges were treated as undermining the ability to use or weigh information relevant to sexual relations. This raises broader concerns about whether findings of incapacity are becoming influenced by the seriousness of perceived safeguarding risks, particularly in cases involving sexual conduct and the potential harm to others.

The Court of Appeal rejected the approach of HHJ Burrows at first instance. Baker J identified three fundamental errors in the first instance reasoning and allowed the appeal on grounds one and two. First, HHJ Burrows had failed to establish a clear causative nexus between ZX’s inability to use or weigh relevant information and his mental disorder, being caused by a disturbance in the functioning of the mind or brain as required by section 2(1) of the MCA, finding only a connection between the two.^22^ Baker J held that HHJ Burrows merely finding a connection between the two was insufficient, erroneously lowering the criterion and quality of evidence that is required to rebut the presumption of capacity.^23^ Secondly, HHJ Burrows had based his conclusion in part on ZX’s history of offending, which Baker J held was not by itself indicative of an inability to understand, use, or weigh information about consent. Instead, Baker J states that:

This pattern of conduct is not by itself indicative of an inability to understand, weigh or use information about consent but is consistent with having the ability to understand and use information but choosing not to do so.^24^

Baker J believed that at first instance, HHJ Burrows had not given an adequate explanation as to why a person described as cunning, opportunistic, and capable of planning sexual contact was nonetheless unable to understand or weigh information about consent.^25^

Critically, Baker J also found that the reliability of Dr Ince’s opinion had been undermined by his misreading of *Re ZZ* as having changed the law on the relevant standard of sexual capacity, a misreading that HHJ Burrows had not adequately interrogated despite acknowledging it.^26^ The case was thus remitted to Theis J for a fresh capacity assessment conducted on the principles of *JB*.

### B. Overlapping domains and safeguarding after remittal

Following remittal, further hearings occurred in May and June 2025.^27^ The proceedings were heard in private due to the unusual facts and the risks of jigsaw identification.^28^ As a result of the conclusions in Dr Williams’ reports at the hearing on 17 June 2025, the court made final declarations that ZX lacked the capacity to make various decisions. These included where to live, care, use of the Internet and social media and managing his property, amongst others.^29^ Yet, the court further declared that ZX had the capacity to decide whether to engage in sexual relations. However, directions were made that there was a need for further assessment in relation to ZX’s capacity to decide about having contact with others, given the lack of clarity on the issue in Dr Williams’ reports and ZX’s taking of unusual risks with his own safety to meet people.^30^

Consequently, a further hearing occurred in December 2025, where Dr Williams gave further oral evidence assessing ZX’s capacity to have contact with others. It was this hearing that the 2026 judgment considered. Dr Williams acknowledged that ZX’s capacity in this area was complex and finely balanced.^31^ She identified that for ZX, a key driver of his behaviour and part of the relevant information in assessing his capacity for contact is his desire to forge new relationships in person via online interaction.^32^ The evidence revealed ‘that ZX does not reflect in practice on what risks might emerge out of in person contact with casual acquaintances’.^33^

Further, Dr Williams considered that ZX’s incapacity to have contact with others was caused not only by ZX’s traumatic history but also by his learning difficulty, stating ‘his history has left a gap in his skills, but this is exacerbated by his learning difficulty … in the moment he is unable to use or weigh the information’.^34^ Furthermore, Dr Williams recognized that the complexity in the assessment process arose from difficulties in determining whether ZX is able to use and weigh the relevant information in the moment when making decisions about contact.^35^ The court accepted Dr Williams’ evidence that ZX would struggle to problem-solve in the moment, and this was largely due to his learning difficulty.^36^ The court also accepted that this extended to sexting and online communications, which Dr Williams argued should be viewed through the prism of contact. The parties agreed it was open to the court to conclude that ZX lacked capacity to have contact with others, and on hearing and agreeing with Dr Williams’ testimony, the court so declared that ZX did not have capacity to have contact with others.

It is this declaration, read alongside the June 2025 finding that ZX has the capacity to engage in sexual relations, that generates the central doctrinal tension this commentary examines. Dr Williams herself acknowledged the difficulty of disentangling the domains of sexual relations, contact with others, and internet use, recognizing that the relevant information overlaps substantially across all three.^37^ The functional approach of the MCA required each to be assessed and declared upon separately. Whether this framework is adequate to the task of producing fair and coherent outcomes where domains are this closely related is the question to which this commentary now turns.

## IV. Critical issues

### A. Siloing decisions and contradictory capacities

The decision in *ZX* reveals that even in cases where Ps are deemed to hold sexual capacity, they can still lack capacity to have contact with others and so are subject to restrictions on their liberty. Unlike sexual capacity, capacity for contact with others may be person-specific and requires P to be able to assess the risk that contact with the other person poses. Therefore, different approaches to capacity assessments are applied in these seemingly similar domains.^38^ Indeed, the decision-specific functional approach of the MCA seeks to avoid status-based paternalism and preserve autonomy through assessing distinct decisions. However, the functional approach may create disjointed assessments and impose autonomy restrictions where capacity decisions overlap.

As noted above, within *ZX*, Dr Williams stated that when assessing ZX’s capacity, she found it difficult to disentangle the overlapping capacities and recognized that ‘in these categories much of the relevant information overlaps’.^39^ As such, the Court’s application of the MCA’s functional structure may misrepresent how decision-making actually occurs. Indeed, ZX’s impulsivity spreads across different but interrelated decision-making domains such as contact, social media, and sexual relations, and yet the court treated these separately. This therefore risks a fragmented understanding of situational decision-making. Consequently, the application of the MCA can be criticized for encouraging micro-decision making, in which complex lives and decisions are reduced to individual legal silos.

The MCA and the approach of the Court of Protection therefore assume decisions can be neatly isolated but fail to consider that cognition is often comprehensive and context-dependent, highlighting how the functional test of the MCA can become overly formalistic. This approach requires focusing on discrete cognitive abilities rather than considering real-world decision-making capacity. These problems are brought to light in cases like *ZX*, where it made little sense to deem ZX to lack the capacity to decide to have contact with others due to difficulty in assessing risk, but, in the same breath, to deem ZX as able to engage in sexual relations with other people. This is particularly problematic as, when both situations involve the weighing of similar issues, the difficulties with using and weighing information in one context are probably transferable across others. As such, the functional approach of the MCA, with its requirement to silo decision-making, risks a legal approach detached from psychological reality.

This division of decision-making capacity can therefore produce inconsistent or contradictory outcomes and can undermine the coherence of the Court of Protection’s approach in sexual capacity or contact cases. By treating these interrelated domains separately under the functional approach, the Court of Protection may mistakenly create safeguarding gaps, underestimate vulnerability, or impose restrictions within areas of autonomous decision-making.^40^

Nevertheless, the MCA’s approach should not be completely dismissed. Without silos, the courts risk reverting to status-based judgements, which the MCA was designed to eliminate. The drawbacks of such an approach may therefore lie in the tendency to strictly assess capacity in isolation for each individual decision, even in cases where decision-making domains are clearly interrelated and interdependent.

Subsequently, a more satisfactory method may lie in developing a refined, context-sensitive interpretation of the MCA that better accommodates overlapping capacity. As several academics have noted, capacity should be understood as inherently relational and shaped by the context in which decisions arise, rather than as a series of isolated cognitive decisions.^41^ This does not require abandoning the decision-specific framework altogether but rather adapting it to recognize when domains are inherently interdependent and therefore require a more comprehensive analysis. In this respect, *ZX* highlights the need for an evolution in how the MCA is interpreted, one that more accurately reflects the interrelated nature of real-world decisions and capacity.

### B. Taking a protective role

A further difficulty exposed by *ZX* lies in the way that the MCA responds to situations in which P poses a risk to others, particularly in sexual contexts. On its enactment, the MCA was described as a visionary piece of legislation, being primarily designed to safeguard P.^42^ Subsequent practice has seen the MCA’s framework, particularly findings of incapacity and the use of the best interests, used indirectly to manage risk. Concerns about risk are heavily ingrained in the MCA jurisprudence.^43^ Where concerns arise about harmful sexual behaviour or exploitative relationships, the court is required to navigate a tension between respecting P’s autonomy and preventing harm to others. As a result, the MCA may be deployed in ways that extend beyond its intended purpose, raising the possibility of overprotective outcomes.

Before advancing this critique, it is necessary to highlight that risk is not an alien intrusion into capacity assessments, but is rather an inevitable and principled feature of the MCA. The relevant information that P must be able to understand, retain, use, and weigh necessarily includes information about consequences, which inherently involve risk. In *JB*, the Supreme Court held that the relevant information for capacity to engage in sexual relations includes the fact that the other person has the right to refuse or withdraw consent and that this decision must be respected.^44^ This means that the courts cannot ignore real risks of harm to third parties, particularly where those risks arise in sexual contexts involving vulnerable individuals.

It is important, however, to maintain the distinction between a finding of incapacity and a best interests determination. Risk to third parties may legitimately inform the latter, and the court may conclude it is not in P’s best interests to act in ways that harm others. However, it cannot be a substitute for the former. A finding of incapacity requires an established inability to make a decision because of a mental impairment; it is not a welfare or protective mechanism, however pressing the protective concern.

The first instance judgment reveals where risk begins to drive the reasoning. HHJ Burrows placed significant weight on ZX’s history of sexually harmful behaviour in concluding that he lacked the capacity to engage in sexual relations. As noted above, no adequate explanation had been given as to why a person described as cunning, opportunistic, and capable of planning sexual contact was nonetheless unable to understand or weigh information about consent.^45^ This is precisely the moment at which risk crossed from relevant information into outcome-determination, where ZX’s dangerousness, rather than his functional decision-making ability, was doing the work of displacing the presumption of capacity. The Court of Appeal corrected this mistake, as Baker J was clear that ZX’s history of non-consensual behaviour was consistent with his choosing not to respect consent, not with an inability to understand or weigh the relevant information. However, the significance of the remitted hearing is not that it resolves the tension exposed at first instance but that even within a methodologically careful application of the correct test, the same structural difficulty reasserts itself.

On remittal, Dr Williams’ assessment focused heavily on ZX’s inability to use and weigh relevant information in the moment. She maintained that this was caused by his learning difficulty and that the inability to problem solve was driven by the learning difficulty specifically.^46^ Theis J also explicitly acknowledged that many individuals who have suffered abuse and lived in care may be vulnerable, but that they do not fall within the jurisdiction of the Court of Protection unless there is evidence of an inability to make decisions because of an impairment, a reminder that welfare concerns cannot substitute the functional test.^47^ The argument this commentary advances, therefore, is not that the court erred. It is that even within that careful framework, the structure of the assessment, the relevant information, the examples used to ground incapacity, and the acknowledged impossibility of disentangling overlapping domains reveal a deeper tension within the structure and subsequent application of the MCA.

Dr Williams’ framing is notable in a further respect. As mentioned earlier, she identified that for ZX, a key driver of his behaviour was his desire to forge new relationships in person via online interaction. The relevant information in a contact capacity assessment is ordinarily concerned with the nature of the relationships and the risks the contact poses to P.^48^ The inclusion of ZX’s relational desires as part of the information he cannot properly weigh is a more expansive formulation, one that incorporates not only what ZX needs to understand about contact, but also what motivates his contact and where those motivations lead. This expansion is understandable given ZX’s profile, but it brings the assessment closer to evaluating whether ZX’s contact decisions are safe ones rather than whether he can process the relevant information about them. The line between those two inquiries is precisely what section 1(4) MCA is designed to maintain,^49^ and the judgment does not explain how that line is preserved when motivation and outcome are folded into the relevant information itself.

This structural concern is reinforced by the three examples used in the contact capacity assessment to illustrate ZX’s inability to use and weigh relevant information: absconding to meet someone online, messaging a younger resident, and contacting a former support worker. Each of these is an example of a risky or harmful outcome of ZX making contact that endangered others or breached suitable boundaries. None of them straightforwardly demonstrates that ZX was unable to process the relevant information about contact at the time. The blurring here is subtle but significant. It is here that the risk critique and the silo critique of this commentary converge: the framework of contact capacity assessments achieves what the sexual relations framework cannot and effectively regulates the sexual risk ZX poses under the formal guise of a different incapacity finding.

This is what makes the structural problem exposed by *ZX* deeper than mere judicial error. The MCA is capable of preserving formal autonomy while simultaneously enabling substantial protective control. It grants capacity with one hand, only to remove its practical conditions with the other. The decision-specific framework, designed as a safeguard against overreach, becomes, in cases like *ZX*, the very mechanism through which overreach is achieved because it permits risk to migrate between domains.^50^ As Reed-Berendt and Clough have observed, the ongoing regulation of disabled people’s sexuality and relationships through the MCA framework is not a neutral exercise.^51^ Findings of incapacity in cases involving individuals who pose sexual risks carry consequences for autonomy that are not captured by the formal framework of the individual declaration.^52^ Greater doctrinal clarity is therefore needed not only to ensure that individual capacity assessments remain genuinely functional in their reasoning but also to require that the cumulative effect of declarations across interconnected domains is capable of scrutiny as a unified question of proportionality, particularly where, as in *ZX*, the combined effect of those declarations engages P’s rights under Article 8 of the European Convention on Human Rights in the most intimate aspects of his life.

## V. Conclusion

*ZX* exposes two structural faults in the MCA’s approach to sexual capacity. The decision-specific framework, designed to protect autonomy to avoid status-based findings, produces fragmented assessments that can generate contradictory outcomes. It is capable of declaring capacity for sexual relations while simultaneously restricting the practical conditions that make that capacity meaningful. In cases where P poses a genuine risk to others, the same framework enables risk to migrate between capacity domains without that migration appearing as a substantive legal error.

These are not mere problems of judicial oversight. The first instance reasoning in *ZX* was corrected on appeal, and Theis J’s approach on remittal was careful. The problems are structural: they arise from the combination of the MCA’s siloed assessment framework and its inability to deliver transparent scrutiny of the cumulative liberty-restricting effect of declarations across interlinked domains. A more relational and context-sensitive interpretation of capacity, one that asks more explicitly when domains are too interdependent to be assessed in isolation, would represent a more meaningful step toward coherence.

## Notes

1 [2021] UKSC 52.

2 ibid [121].

3 ibid.

4 [2026] EWCOP 3.

5 Mental Capacity Act 2005.

6 ibid, s 2.

7
*A Local Authority v ZX* [2024] EWCOP 30.

8 ibid [14].

9 ibid [26].

10 ibid [16–17].

11 ibid [22].

12 ibid [24–25].

13 ibid [66].

14
*ZX* (2026) (n 4) [18].

15 ibid.

16 ibid.

17
*ZX* (2024) (n20) [113].

18 ibid [113].

19 ibid [114(8)].

20 ibid [110].

21 ibid [115].

22
*ZX (Capacity to Engage in Sexual Relations)* [2024] EWCA Civ 1462 [66].

23 ibid.

24 ibid [65].

25 ibid.

26 ibid [60].

27
*ZX* (2026) (n4).

28 ibid [2].

29 ibid [6].

30 ibid [7].

31 ibid [32].

32 ibid [17].

33 ibid [17].

34 ibid [21].

35 ibid [33].

36 ibid [33].

37 ibid [16].

38 [2013] EWCA Civ 478.

39
*ZX* (2026) (n4) [16].

40 Emma M Szelepet, ‘The Mental Capacity Act 2005 Best Interests Test as Applied to the Elderly – Is It Fit for Purpose?’ (2018) 86 Medico-Legal Journal 142.

41 Deborah O’Connor, ‘Personhood and Dementia: Toward a Relational Framework for Assessing Decisional Capacity’ (2010) 5 Journal of Mental Health Training, Education and Practice 22; Camillia Kong, *Mental Capacity in Relationship: Decision-Making, Dialogue, and Autonomy* (CUP 2017); Jonathan Lewis, ‘Re-Envisioning Autonomy: From Consent and Cognitive Capacity to Embodied, Relational, and Authentic Selfhood’ (2025) 20 Clinical Ethics 1.

42 House of Lords Select Committee on the Mental Capacity Act 2005, Mental Capacity Act 2005: Post-Legislative Scrutiny (HL Paper 139, 2014).

43 John Fanning, ‘Continuities of Risk in the Era of the Mental Capacity Act’ (2016) 24 Medical Law Review 415.

44
*JB* (n1) [121].

45
*ZX* (2024) (n39) [65].

46
*ZX* (2026) (n4) [21].

47 ibid [30].

48 See *Re B* (Capacity: Social Media: Care and Contact) [2019] EWCOP 3;  *Lincolnshire County Council v AB* [2019] EWCOP 43; *Hull City Council v KF* [2022] EWCOP 33.

49 MCA (n5).

50 Lucy Series, ‘Relationships, Autonomy and Legal Capacity: Mental Capacity and Support Paradigms’ (2015) 40 International Journal of Law and Psychiatry 80.

51 Ruby Reed-Berendt and Beverley Clough, ‘(Un)blurred Lines? Sex, Disability, and the Dynamic Boundaries of Mental Capacity Law’ (2024) 93 International Journal of Law and Psychiatry 101960.

52 ibid.

